# Editorial: For The Future of Alkaliphiles: 50th Anniversary Year Since the Rediscovery of Alkaliphiles by Dr. Koki Horikoshi

**DOI:** 10.3389/fmicb.2019.02017

**Published:** 2019-09-03

**Authors:** Masahiro Ito, Terry Ann Krulwich

**Affiliations:** ^1^Graduate School of Life Sciences, Toyo University, Gunma, Japan; ^2^Icahn School of Medicine at Mount Sinai, New York, NY, United States

**Keywords:** alkaliphiles, Na^+^/H^+^ antiporter, alkaliphilicity, ESKAPE pathogens, alkaliphilic streptomyces, indigo-reduced microorganisms, the candidate phylum NPL-UPA2, the cedars

Dr. Koki Horikoshi, one of the founders of the International Society for Extremophiles (ISE), its first president and founding editor of the journal Extremophiles, passed away on the 16th of March 2016. Dr. Horikoshi devoted his time as a researcher to understanding the molecular basis for microbial survival under extreme conditions and leaves behind an enduring legacy as a pioneer of extremophile research. He is particularly famous for rediscovering alkaliphiles (alkaline-loving microorganisms), for leading multiple studies on their physiology and adaptive mechanisms, and for successfully industrializing a number of alkaliphilic enzymes. Dr. Horikoshi was a consummate academic and leader, deeply devoted to the development of research institutions such as the renowned Japanese Agency for Marine-Earth Science and Technology (JAMSTEC). He was also a highly personable man, dedicated to his family, country and the mentoring of young academics. Thanks to his immense contributions, Japan is a global leader in several areas of extremophile research, including alkaliphile and hyperthermophile microbiology.

Dr. Horikoshi received several awards for his scientific contributions, including the prestigious Medal of Honor with Purple Ribbon from the Japanese Government (1987), the Gold Medal from the International Institute of Biotechnology by Prince Michael of Kent at the Royal Society, London (1991), the Honda Prize (1993), and the Japan Academy Prize (2006).

The first encounter between Dr. Horikoshi and alkaliphiles dates back 50 years (Horikoshi and Akiba, 1982; Horikoshi, 2016). At the end of October 1968, he visited Florence in Italy and the sight of the Renaissance architecture, so different from that of Japan, helped to develop and crystallize his ideas regarding unknown microorganisms living in different extreme environments.

When he returned to Japan, he initiated a new program of research on alkaliphilic organisms. There followed nearly 50 years of research of alkaliphilic microbiology during which time over two thousand relevant research papers have been published. Dr. Horikoshi and his coworkers have made substantial contributions in this field and their pioneering work has established a solid baseline for exploring the molecular basis of alkaliphilic adaptation.

It is because of this vast legacy of research that we decided to honor Dr. Horikoshi by assembling a Research Topic on alkaliphilic microbiology. These articles cover a wide range of topics and provide new insights into alkaliphilicity.

Aino et al. review structural changes in bacterial communities during indigo fermentations, which occur under alkaline anaerobic conditions, and discuss the stability of the microflora. The authors consider the role of the microflora and how diversity plays an important role in maintaining the reduced state of long-term indigo fermentation. The second review by Matsuno et al. focuses on Mitchell's chemiosmotic theory and the inconsistencies of ATP production of alkaliphiles in highly alkaline environments. Several variations on efficient ATP production and adaptation of bacteria to alkaline environments are noted. Finally, the authors discuss the cytochrome c-related “H^+^ capacitor mechanism” is as an alkaline adaptation strategy. The third review by the Ito et al. evaluates our understanding of bacterial and archaeal Mrp-type Na^+^/H^+^ antiporters. The authors consider the ion transport pathway of Mrp, which is known to play an important role in pathogens. The primary article by Suzuki et al. presents interesting results from the first metagenome assembled genome (MAG) and *in-situ* gene expression data of the candidate phylum NPL-UPA2 in a serpentinization site called The Cedars. Terra et al. investigated the ethnopharmacological healing of alkaline/radon in soils from the Boho region of Northern Ireland and isolated a new *Streptomyces* sp. which grew at high alkaline pH and was resistant to gamma radiation. *In vitro* testing of isolates also yielded important results in inhibiting ESKAPE pathogens. The research article by Takahashi et al. showed that BpOF4_01690, a monocistronic small hydrophobic protein of the alkaliphilic microorganism *Bacillus pseudofirmus*, plays an essential role in oxidative phosphorylation under highly alkaline conditions. Yang et al. generated chimeras of the NhaD-type Na^+^/H^+^ antiporters NhaD1 and NhaD2 of halotolerant and alkaliphilic *Halomonas* sp. Y2 and demonstrated functional changes and responses to pH. The studies by Fujinami and Ito both dealt with CsaB-deficient mutants involved in the anchoring of S-layer homology (SLH) domain-containing proteins of alkaliphilic *Bacillus pseudofirmus* to the cell surface and investigated cell surface proteins and the mechanism of “alkaliphilicity.”

## Author Contributions

MI and TK are co-editors of the Research Topic and discussed the writing.

### Conflict of Interest Statement

The authors declare that the research was conducted in the absence of any commercial or financial relationships that could be construed as a potential conflict of interest.

## References

[B1] Horikoshi K. (2016). Chapter 4 “Alkaliphilesm,” in Extremophiles, Where it All Began (Tokyo: Springer), 53–55. 10.1007/978-4-4321-55408-0

[B2] Horikoshi K. Akiba T. (1982). Preface, in Alkalophilic Microorganisms, a New Microbial World (Berlin, Heidelberg, NY: Springer-Verlag).

